# Supplementary material for: A unifying model for the propagation of prion proteins in yeast brings insight into the [PSI+] prion
Source: PLoS Comput Biol. 2020 May 26;16(5):e1007647. doi: 10.1371/journal.pcbi.1007647 (PMC7274466; doi:10.1371/journal.pcbi.1007647)
Supplement: S1 Text — (PDF) [file pcbi.1007647.s001.pdf]

S1 Appendix - Analytical study of the bi-stable system for prion replication

Paul Lemarre, Laurent Pujo-Menjouet, Suzanne S. Sindi

1 Without impulsions - ordinary differential equations

We start by studying the model without impulsions, given by the following system of ordinary differential equations (ODEs)

$$\begin{aligned} \frac{dV}{dt}(t) =& \lambda - \gamma V - \rho V f(S), \\ \frac{dS}{dt}(t) =& \rho V f(S) - \gamma S. \end{aligned} \tag{1.1}$$

The function f is defined as

$$f(S) = \frac{S^n}{K^n + S^n}, (n > 1). \tag{1.2}$$

Prion-free equilibrium. One equilibrium always exists, it is the prion-free equilibrium $V = \frac{\lambda}{\gamma}, S = 0$. It is straightforward to establish that the Jacobian matrix of the system at this point is

$$\mathcal{J}(\frac{\lambda}{\gamma}, 0) = \begin{pmatrix} -\gamma - \rho f(0) & -\rho\frac{\lambda}{\gamma} f'(0) \\ \rho f(0) & \rho\frac{\lambda}{\gamma} f'(0) - \gamma \end{pmatrix}.$$

Our choice of non-linearity f ensures $f(0) = f'(0) = 0$, so that the Jacobian at the prion-free equilibrium has a double eigenvalue $-\gamma$. This equilibrium is thus locally stable for any choice of (positive) parameters. This shows how introducing a non-linearity of this type creates multi-stability in the model.

Prion equilibria. Two other equilibria can exist with $S \neq 0$. The equilibrium conditions lead to

$$\begin{aligned} V &= \frac{\lambda}{\gamma} - S, \\ \rho(\frac{\lambda}{\gamma} - S) f(S) &= \gamma S. \end{aligned}$$

The choice of f (in particular the conditions $f(0) = 0$,$f'(0) = 0$, and f has a single inflection point) ensures that the equation on S has 0, 1 or 2 positive solutions. The Jacobian of the system is

$$\mathcal{J}(V, S) = \begin{pmatrix} -\gamma - \rho f(S) & -\rho V f'(S) \\ \rho f(S) & \rho V f'(S) - \gamma \end{pmatrix}.$$

If we define $H(S) = \rho(\frac{\lambda}{\gamma} - S)f(S)$, we notice that an equilibrium corresponds to $H(S) = \gamma S$. The function H is non-negative for $S \in [0, \frac{\lambda}{\gamma}]$, has a single maximum point and cancels in both $S = 0$ and $S = \frac{\lambda}{\gamma}$. This tells us that it crosses the line γS up to two times S_1 and S_2, with $0 < S_1 < S_2$. Since $H(0) = H'(0) = 0$, the first crossing is from below. In other words $H'(S_1) > \gamma$, and thus necessarily $H'(S_2) < \gamma$. Now notice that $H'(S) = \rho(\frac{\lambda}{\gamma} - S)f'(S) - \rho f(S)$. With a few rearrangements, the characteristic polynomial of the Jacobian writes as

$$\chi^2 + \chi(2\gamma - H'(S)) + \gamma(\gamma - H'(S)) = 0.$$

The solutions to $\chi = 0$ are the eigenvalues of the Jacobian. Such eigenvalues have negative real parts if and only if both $2\gamma > H'(S)$ and $\gamma > H'(S)$. This gives us directly that S_1 is necessarily unstable because $H'(S_1) > \gamma$. Likewise, we also know that S_2 is stable because $H'(S_2) < \gamma < 2\gamma$. This proves the essential characteristic of this model, when three equilibria exist ($S = 0, S = S_1$ and $S = S_2$) two of them are locally stable, and they are separated by an unstable one. This shows that the prion equilibria appear with a saddle-node bifurcation. The point of bifurcation is not analytically tractable in general (it is for $n = 2$, but we use $n = 5$ in the simulations).

Mass equilibrium. It is useful for later developments to study the behavior of the mass of the system. The mass can be defined as $M = V + S$, the total concentration of protein in the system. It is straightforward to check that

$$\frac{dM}{dt} = \lambda - \gamma M.$$

This equation is solved analytically by $M(t) = \frac{\lambda}{\gamma} + (M(0) - \frac{\lambda}{\gamma})e^{-\gamma t}$. The mass in the system is at equilibrium when $V + S = \frac{\lambda}{\gamma}$.

2 With impulsions - full system

Periodic impulsions. The main simplification used when adding impulsions is to only consider periodic impulsions. With an impulsion time T and an impulsion distribution rate α, the system becomes

$$\begin{aligned}
\frac{dV}{dt}(t) =& \lambda - \gamma V - \rho V f(S), \\
\frac{dS}{dt}(t) =& \rho V f(S) - \gamma S, t \neq kT, \\
V(kT^+) =& V(kT^-), \\
S(kT^+) =& (1 + \alpha)S(kT^-), k \in \mathbb{N}.
\end{aligned}$$

Note that the concentration of monomers is not affected by impulsions because we assume monomers diffuse fast enough at the moment of cell division.

Prion-free solution. This system admits a prion-free solution $V = \frac{\lambda}{\gamma}, S = 0$ (just as the non-impulsive system).

Prion periodic solution. It can also exhibit positive periodic solutions. Such a solution requires

$$\begin{aligned} V(0) =& V(T), \\ S(0) =& (1+\alpha)S(T). \end{aligned}$$

Using the equation on the mass, we can find a more constraining relation between $V(0)$ and $S(0)$. Indeed the mass follows the dynamics given analytically by $M(t) = \frac{\lambda}{\gamma} + (M(0) - \frac{\lambda}{\gamma})e^{-\gamma t}$. In particular, we have

$$M(T) = \frac{\lambda}{\gamma} + (M(0) - \frac{\lambda}{\gamma})e^{-\gamma T}.$$

By using the definition $M(0) = V(0) + S(0)$ and $M(T) = V(T) + S(T)$ as well as the periodic solution conditions, we show that if $V(0) = V(T)$ and $S(0) = (1+\alpha)S(T)$ then

$$V(0) + \frac{1/(1+\alpha) - e^{-\gamma T}}{1 - e^{-\gamma T}} S(0) = \frac{\lambda}{\gamma}.$$

We have a similar linear relationship between $V(T)$ and $S(T)$

$$V(T) + \frac{1 - (1+\alpha)e^{-\gamma T}}{1 - e^{-\gamma T}} S(T) = \frac{\lambda}{\gamma}.$$

This means that a periodic solution will necessarily join those two lines in the phase plan.

This condition is also sufficient. If we find a solution where $(V(0), S(0))$ is on the first line and $(V(T), S(T))$ on the second one, then this solution is a periodic solution for the impulsive system. It is also a direct consequence of the explicit solution for the mass equation. This gives us a reduced space where to look for periodic solutions in the phase plan, and facilitates the numerical determination of periodic solutions.

Saddle-node bifurcation and local stability. No proof is yet suggested in this preliminary work, but we have numerical evidence that the properties of the non-impulsive ODE system are transferred onto the periodic impulsive systems. In particular, the prion-free solution remains locally stable for any choice of parameters, as long as the impulsion remains physically realistic. In the notations of the main text, this is expressed in the assumption $-\pi < \varepsilon < 1 - \pi$. Two prion periodic solutions (with non-zero values of S) appear in a saddle-node bifurcation, one being unstable and the other locally stable. Proof of those properties is planned for future, more theoretical work.
